# Neural Correlates of Empathy with Pain Show Habituation Effects. An fMRI Study

**DOI:** 10.1371/journal.pone.0137056

**Published:** 2015-08-28

**Authors:** Mira A. Preis, Birgit Kröner-Herwig, Carsten Schmidt-Samoa, Peter Dechent, Antonia Barke

**Affiliations:** 1 Georg-August University of Goettingen, Georg-Elias-Mueller Institute of Psychology, Department of Clinical Psychology and Psychotherapy, Goßlerstraße 14, 37073, Goettingen, Germany; 2 University Medical Center Goettingen, Department of Cognitive Neurology, MR Research in Neurology and Psychiatry, Robert-Koch-Straße 40, 37075, Göttingen, Germany; 3 Philipps University of Marburg, Department of Clinical Psychology and Psychotherapy, Gutenbergstraße 18, 35032, Marburg, Germany; University G. d'Annunzio, ITALY

## Abstract

**Background:**

Neuroimaging studies have demonstrated that the actual experience of pain and the perception of another person in pain share common neural substrates, including the bilateral anterior insular cortex and the anterior midcingulate cortex. As many fMRI studies include the exposure of participants to repeated, similar stimuli, we examined whether empathic neural responses were affected by habituation and whether the participants' prior pain experience influenced these habituation effects.

**Method:**

In 128 trials (four runs), 62 participants (31 women, 23.0 ± 4.2 years) were shown pictures of hands exposed to painful pressure (pain pictures) and unexposed (neutral pictures). After each trial, the participants rated the pain of the model. Prior to the experiment, participants were either exposed to the same pain stimulus (pain exposure group) or not (touch exposure group). In order to assess possible habituation effects, linear changes in the strength of the BOLD response to the pain pictures (relative to the neutral pictures) and in the ratings of the model’s pain were evaluated across the four runs.

**Results:**

Although the ratings of the model’s pain remained constant over time, we found neural habituation in the bilateral anterior/midinsular cortex, the posterior midcingulate extending to dorsal posterior cingulate cortex, the supplementary motor area, the cerebellum, the right inferior parietal lobule, and the left superior frontal gyrus, stretching to the pregenual anterior cingulate cortex. The participant’s prior pain experience did neither affect their ratings of the model’s pain nor their maintenance of BOLD activity in areas associated with empathy. Interestingly, participants with high trait personal distress and fantasy tended to show less habituation in the anterior insula.

**Conclusion:**

Neural structures showed a decrease of the BOLD signal, indicating habituation over the course of 45 minutes. This can be interpreted as a neuronal mechanism responding to the repeated exposure to pain depictions, which may be regarded as functional in a range of contexts.

## Introduction

Empathy refers to the capacity to identify with, and vicariously share, the feelings and thoughts of others [1]. According to the perception-action model [2], the perception of actions and emotions activates the same neural mechanisms that are responsible for the actual generation of those actions and emotions. This is supported by a growing number of functional magnetic resonance imaging (fMRI) studies, which demonstrate that the perception of another person in a painful situation leads to activation in regions belonging to the pain matrix, including the bilateral anterior insular cortex (AI) and the anterior midcingulate cortex (aMCC) ([3,4] for reviews).

The phenomenon of habituation is characterised by a decrement of the response to repeatedly presented similar stimuli and is one of the most fundamental forms of nervous system plasticity [5,6,7]. Habituation of behaviour is well documented [8] and there is a growing body of studies investigating habituation with regard to neural activity, i.e., decrement of the BOLD response due to a repeatedly presented stimulus, e.g., [9,10,11]. Since it provides a mechanism for allocating attentional resources to novel stimuli, which have unknown valence, over familiar stimuli, habituation is adaptive [11]. With regard to empathy for pain, the adaptive advantage of habituating to the pain of others after repeated confrontations with a particular type of pain might be the conservation of the observer’s resources when he/she cannot do anything about the pain. Incidentally, these processes are helpful for, e.g., health care professionals when helping patients. In the majority of fMRI studies, the participants are exposed to repeated, often very similar, stimuli. Hence, their neural responses may be influenced by habituation effects. To our knowledge, research concerning the neural correlates of the habituation of empathy over time is lacking. Lamm and colleagues [12] are the only ones who have studied habituation effects on behavioural measures of empathy (ratings of perceived pain), but they found no habituation effects in response to repeated exposure to similar stimuli. However, there is evidence that empathy can be modulated by top-down and bottom-up mechanisms [4,13,14], which can be interpreted in relation to possible habituation effects. For instance, Cheng et al. [15] showed visual stimuli depicting needles being inserted into different body parts to physicians who practice acupuncture and to acupuncture-naïve controls and found the expected signal increase in the insula and aMCC in participants of the control group, but not in participants who were experts in acupuncture. Instead, the experts showed increased activity of the medial and superior prefrontal cortices and the temporoparietal junction, which are involved in emotion regulation and theory of mind. Hence, the familiarity of the pain stimulus may influence habituation of empathy.

The participants’ prior experience may be another mechanism that modulates empathy [16,17,18,19,20]. In our previous study, participants showed increased activity in the AI and aMCC in response to viewing pictures displaying exposure to painful pressure relative to pictures showing no indication of pain. However, participants who had been exposed to the same pain stimulus prior to the experiment showed lower activity in the right AI and the aMCC than participants who did not know what the pain stimulus felt like [21].

Both the study of Cheng and colleagues [15] and of Preis and colleagues [21] examined the influence of familiarity with the pain stimulus on neural correlates of empathy for pain. However, Cheng and colleagues investigated familiarity as it arises from a third person pain experience (in acupuncture experts versus novices), whereas Preis and colleagues experimentally manipulated their participants’ first-hand pain experience. Although both first-hand experience and third person experience resulted in lower aMCC and AI activity, different cognitive mechanisms could have mediated this effect.

The aim of the present analysis was to investigate habituation effects on the neural correlates of empathy and the influence of prior pain experience on these habituation effects. To this end we reanalysed data from our previous study [21]. For both groups, we expected the activation in the aMCC and AI to decrease over time. To our knowledge, prior pain exposure has not yet been investigated with regard to habituation effects. However, the participants with prior first-hand pain experience showed attenuated activations in the AI and aMCC for the pain pictures [21]; since it is characteristic of habituation that reactions to less intense stimuli decrease more rapidly and/or more pronouncedly [8,22], one could hypothesise that the experienced group’s attenuated activations in response to the pain pictures would represent perhaps a habituation process.

## Material and Methods

### Ethics Statement

Ethical approval was obtained from the Ethics Committee of the Georg-Elias-Mueller Institute for Psychology at the University of Goettingen (Germany); all participants gave their written informed consent before their participation.

### Sample

We reanalysed data collected in the experiment described in detail in Preis et al. [21]. A total of 64 healthy, right-handed Caucasian students (32 females, 32 males) participated in that study. For the analysis of habituation effects, data sets of all four runs (see stimulation paradigm) had to be complete. This resulted in the exclusion of two participants (one female: run 3 incomplete, one male: run 4 incomplete) from that study. The final sample thus included 62 participants (31 females, 31 males) aged between 19 and 37 years (23.0 ± 4.2 years).

The participants, who were recruited from undergraduate classes at the University of Goettingen, were either paid or received course credits for their participation. In addition, each participant was given a CD with the anatomical pictures of his or her brain. Exclusion criteria were as follows: standard MRI contraindications (e.g., metal objects in the body), left-handedness (assessed with the Edinburgh Handedness Inventory [23]), having a history of neurological or psychiatric disease, suffering from chronic pain, or prior experience in experimentally-induced pain by mechanically applied pressure (assessed using a self-report questionnaire).

### Design of the Study

We used a 2 x 2 repeated-measure design with the between-factor exposure (pain exposure and touch exposure) and the within-factor picture type (pain vs. neutral).

#### Exposure

Prior to scanning, the participants were exposed to either painful pressure (pain exposure group) or a light touch (touch exposure group). The participants were randomly allocated to the conditions (randomisation proceeded separately for men and women in order to achieve a balanced sex ratio). Due to the exclusion of two participants, the pain exposure group included 16 females and 15 males and the touch exposure group 15 females and 16 males.

For the administration of pressure or touch, we used a stationary pressure algometer developed by Goebel and colleagues [24,25], which exerted constant pressure through a plunger with a circular contact area of 2.56 mm^2^. The amount of pressure can be varied with an adjustable weight. An electric motor lowers a plunger at a constant speed at the push of a button and lifts it again after 10 seconds. In the pain exposure condition, the participants were exposed to the pressure stimulus (1.68 MPa) on the middle phalanx of their left forefinger to gain experience with the pain that they would later observe in the model. In the touch exposure group, the pressure cylinder just lightly touched the middle phalanx of the forefinger, thus no pain was induced. The purpose of the touch exposure was to familiarize the participants in this group with the algometer without actually exposing them to the pain itself. This was done to avoid any novelty effects in the group without the pain exposure when observing the pictures of the model during the scanning.

#### Visual stimuli for empathy induction

Sixty-four static colour photographs were presented, each showing the left hand of one of 16 models (the objects of empathy). Each model contributed four pictures (two for each condition). The models (8 female, 8 male) were Caucasian, aged between 22 and 31 years (25.0 ± 2.5 years). All hands were shown from a lateral perspective.

**Fig 1:**
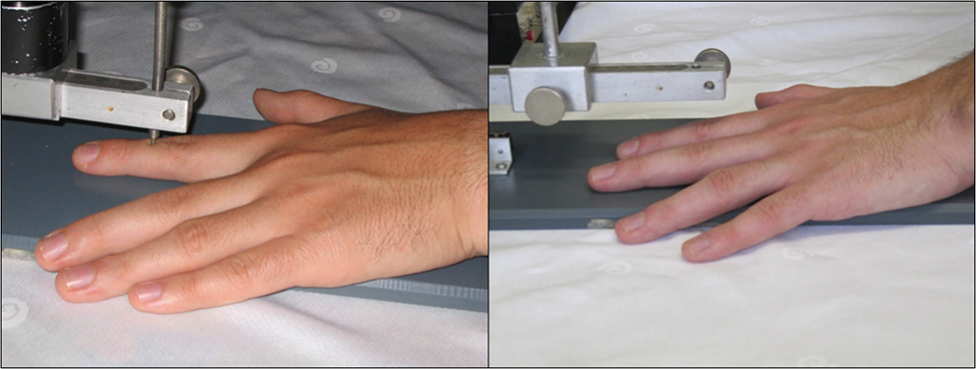
Example of pain picture and neutral picture.

There were two types of pictures (picture type). In 32 pictures, the pressure cylinder of the algometer exerted painful pressure upon the left forefinger (pain pictures). The remaining 32 pictures showed the algometer with the plunger removed, thus showing no pain to the depicted hand (neutral pictures). Apart from this detail, the scenes in the pictures were identical (Fig 1). In order to induce empathic responses, the participants were instructed to imagine how the person in the picture feels and to rate the level of pain felt by the model.

### Assessment of Behavioural Measures

#### Pain intensity

After exposure to pain or touch, the participants rated the intensity of the pain during the application (‘self-pain’) using an 11-point numeric rating scale (NRS; paper and pencil) with the endpoints 0, *no pain at all*, and 10, *worst pain imaginable*.

During the scanner session, the participants rated the pain of the photographed models (‘pain of model’, POM) after each picture on a comparable NRS (computer version) by using three buttons (left; log in; right) with their right (dominant) hand to move the cursor between the two endpoints and to log their rating. The time for the response was limited to six seconds.

#### Trait empathy

The participants’ trait empathy was measured with the German version of the Interpersonal Reactivity Index (IRI [26]), the Saarbruecker Persoenlichkeitsfragebogen (SPF) [Saarbruecker Personality Questionnaire] [27]. The IRI consists of 28 items rated on a 5-point scale with the anchors *does not describe me well* to *describes me very well*. The items are arranged into four subscales with seven items. Each subscale measures a distinct component of empathy: empathic concern (feelings of compassion and concern for others, e.g., “When I see someone being taken advantage of, I feel kind of protective towards them.”); personal distress (feelings of anxiety and discomfort that result from observing another person’s negative experience, e.g., “Being in a tense emotional situation scares me.”); perspective taking (the ability to adopt the perspectives of other people and see things from their point of view, e.g., “I sometimes try to understand my friends better by imagining how things look from their perspective.”); and fantasy scale (the tendency to identify with characters in movies, books, or other fictional situations, e.g., “I really get involved with the feelings of the characters in a novel.”) [26] The internal consistency of the SPF was satisfactory (Cronbach’s α = 0.78; [27]).

### Stimulation Paradigm

**Fig 2:**
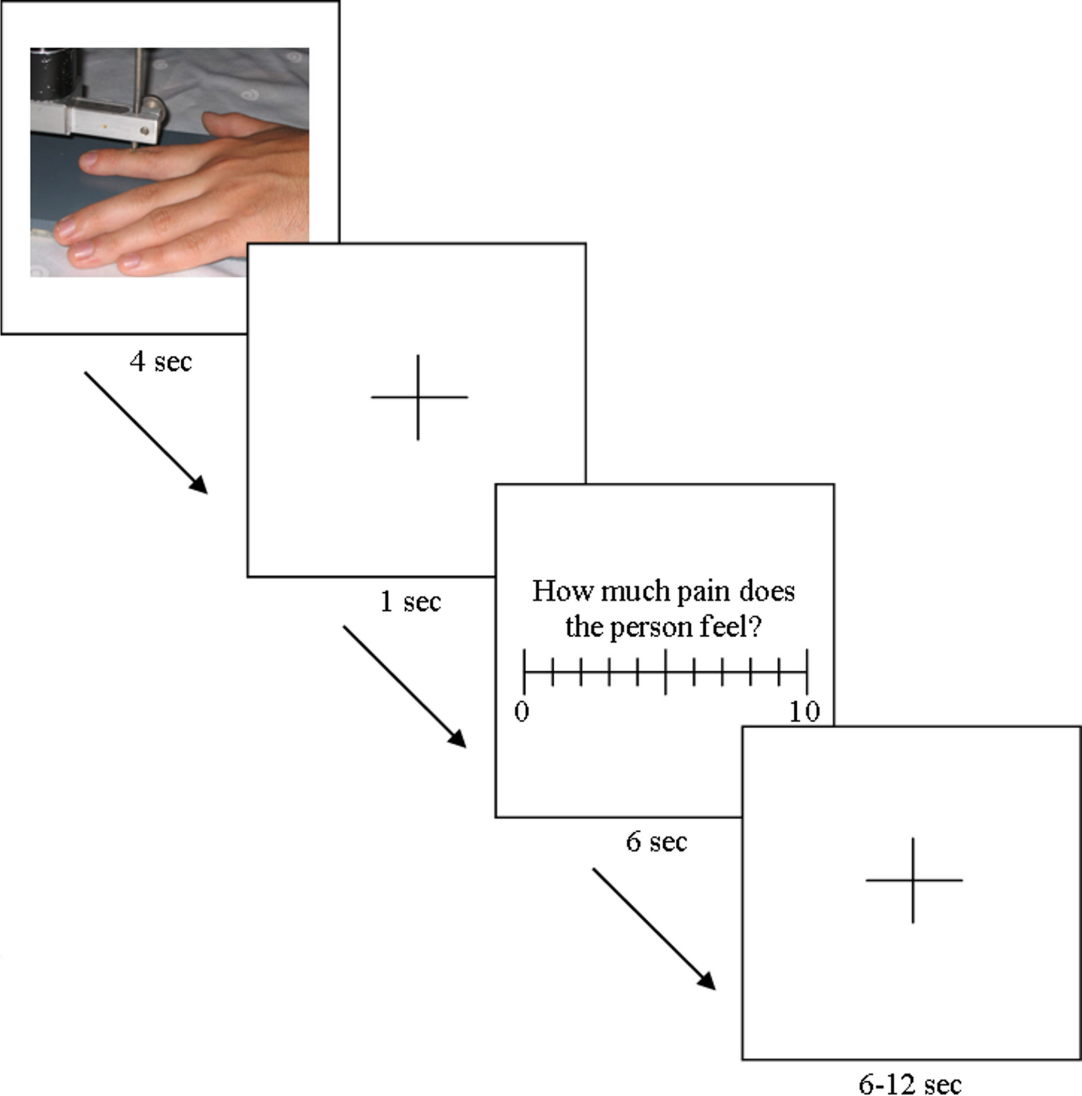
Stimulation paradigm.

Each person participated in 128 trials grouped into four blocks (runs) (32 trials in each run). A trial consisted of the presentation of a picture (pain or neutral) for 4s, followed by a fixation cross (1s). Subsequently, the participant rated the model’s pain (6s time limit). The next trial began after an intertrial interval (white fixation cross on black background), which randomly varied between 6 and 12s (mean duration = 9s). In 50% of the trials a jitter of one second was included (Fig 2). Each run lasted 624s (10.4 min).

### Procedure

The participants completed the SPF [27] and were then seated in front of the pressure algometer. After the setup and the process of pain induction was explained, the participants were instructed to place their left forefinger under the plunger and, depending upon the assigned condition, were exposed to either pain or a light touch. Thereafter, each participant was placed in a supine position in the MRI scanner. After the anatomical reference scans, the functional imaging with the picture presentation proceeded. The pictures were presented using MR-compatible, liquid crystal display goggles with a resolution of 800 x 600 (Resonance Technology, Northridge, CA, USA). Participants who required glasses received corrective lenses, which were combined with the goggles in order to ensure corrected-to-normal vision.

### Image Acquisition

MR imaging was performed at 3 Tesla (Siemens Magnetom TIM Trio, Siemens Healthcare, Erlangen, Germany), using a standard eight-channel phased-array head coil. All participants wore foam earplugs for noise protection and headphones for communication with the experimenter. Initially, an anatomical 3D T1-weighted dataset was acquired (Turbo fast low angle shot (FLASH), echo time (TE): 3.26 ms, repetition time (TR): 2250 ms, inversion time: 900 ms, flip angle 12°), which covered the whole head at 1 x 1 x 1 mm^3^ isotropic resolution. The functional datasets were acquired using T2*-weighted, gradient-echo, echo-planar imaging (TE: 30 ms, TR: 2000 ms, flip angle 70°, 33 slices of 3 mm thickness (20% interslice gap) at an in-plane resolution of 3 x 3 mm^2^ in ascending order). Within one functional run, 312 whole brain volumes were recorded. To account for T1-equilibrium effects, four preparatory scans were acquired and subsequently discarded from the analysis.

### Data Analysis

#### Behavioural data analysis

Behavioural data were analysed using SPSS 21 (SPSS Inc., USA). *T* tests were calculated to test whether the groups with pain exposure and with touch exposure differed with regard to self-pain and trait empathy. We assumed that habituation could be demonstrated as a linear decrease in POM ratings over the four runs. Therefore, for each subject we calculated the mean POM rating per run and estimated the slope of the best-fitting regression line across these four values. The resulting values are called POM slopes. Positive slopes would indicate that the ratings increase over time, whereas negative slopes would express a decrease (habituation). The stronger the habituation the steeper the negative slope (i.e., the smaller the POM slope value). For the whole sample, a one sample *t* test was conducted to investigate whether the POM slopes differed significantly from 0, that is, whether the ratings showed habituation. A two sample *t* test was computed to test whether the pain exposure and the touch exposure group differed with regard to the POM slopes. To test whether trait empathy was related to the degree of habituation, we calculated the correlations of the IRI subscales with the POM slopes. As smaller POM slope values express stronger habituation, a positive correlation between trait empathy and POM slopes would indicate that high trait empathy corresponds to less habituation. The significance level was set at *p* < 0.05.

#### fMRI data analysis

Preprocessing–The functional data analyses were conducted using Brain Voyager QX Software version 2.1.2 (Brain Innovation, Maastricht, The Netherlands). Standard preprocessing steps included 3D motion correction, slice scan time correction, temporal filtering (linear trend removal and high pass filtering (Fourier with 0.0048 Hz as cut-off)) and spatial smoothing with a Gaussian kernel (full width at half maximum 8 x 8 x 8 mm^3^). The functional datasets were coregistered to the anatomical reference scans and transformed into Talairach space. A random effects group analysis was performed on the basis of the multisubject approach of the general linear model. The four seconds of picture presentation were convolved with the canonical haemodynamic response function, which resulted in two different predictors (pain pictures, neutral pictures). The one-second fixation cross presentations after the picture presentation and rating procedures were included in the design matrix as predictors of no interest.

Whole brain analyses–To evaluate habituation effects that are specific for the pain stimuli the following approach was used: For each subject we estimated separate beta values for the two picture types (pain, neutral) in each of the 4 runs using a standard GLM approach. To control for habituation effects that are not specific for the pain stimuli, the run-wise difference between the beta value for pain and no-pain condition was calculated yielding 4 contrast maps per subject, one for each run. We assumed that habituation could be demonstrated as a linear decrease in this beta difference over the runs. At each voxel and for each subject, the slope of the best-fitting regression line was estimated. Using a standard summary statistics approach, we tested at each voxel whether the slopes for the group differed significantly from zero using a one sample *t* test. Positive slopes would indicate that the activation in this area increases over time, whereas negative slopes would express a decrease (habituation). Stronger habituation corresponds to steeper negative slopes (i.e., smaller values of the BOLD slope).

A *t* test between the groups (pain exposure versus touch exposure) was conducted to investigate the influence of prior pain experience on habituation. The slopes of the BOLD response were correlated with POM slopes and trait empathy ratings. As smaller slope values of the BOLD response express stronger habituation, positive correlations between trait empathy ratings and the slopes of the BOLD response would indicate that a high occurrence of this trait corresponds to less neural habituation. As smaller slope values for both the BOLD response and POM ratings express stronger habituation a positive correlation of these variables would indicate that a stronger reduction in POM ratings corresponds to stronger neural habituation.

Corrections for multiple comparisons were performed as follows: Corrections for all contrasts were based on the cluster level estimation [28,29]. The uncorrected threshold was set at *p* = 0.01; Montecarlo simulations (1000 iterations) were performed on the basis of the number of activated voxels and the estimated smoothness of the map to determine the minimum cluster size required to yield an error rate of no more than *p* = 0.05 at the cluster level. Peak activations were identified by the nearest grey matter coordinates in the Talairach Demon database [30,31].

## Results

### Behavioural Data

The groups (pain exposure vs. touch exposure) did not differ with regard to trait empathy (Table 1). The manipulation of exposure resulted in significantly higher self-pain ratings for the pain exposure group (*M* = 3.45, *SD* = 0.32) relative to the touch exposure group (*M* = 0.00, *SD* = 0.00; *t*(60) = 10.76, *p* < .0001). In the pain exposure group, there was a positive correlation between the self-pain ratings and the average POM ratings (*r* = .79, *p* < .0001).

**Table 1:** Means (*M*), standard deviations (*SD*) and differences between the groups (*t*-tests) in age, self-pain ratings, POM ratings and the SPF.

	*Pain exposure condition (n = 31)*		*Touch exposure condition (n = 31)*		*Differences*	
	*Mean*	*SD*	*Mean*	*SD*	*t* (60)	*p*
**Age**	23.13	4.35	22.84	4.05	0.27	.79
**Self-pain**	3.45	0.32	0.00	0.00	10.76	< .0001
**POM**	3.40	1.63	3.22	1.96	0.39	.70
**SPF—Fantasy Scale**	14.00	3.40	13.52	3.46	-0.56	.58
**SPF—Perspective Taking**	14.65	2.30	15.16	2.27	0.90	.38
**SPF—Empathic Concern**	14.39	3.15	15.16	2.57	1.06	.29
**SPF—Personal Distress**	9.81	2.12	9.94	2.93	0.43	.67

*Note*. *POM*, *Pain of model; SPF*, *Saarbruecker Persoenlichkeitsfragebogen*. Self-pain ratings and POM ratings were each measured on 11-point NRSs (0–10).

POM slopes did not differ significantly from zero (*M* = -0.08, *SD* = 0.37; *t*(61) = -1.64, *p* = .11). There was no significant group difference regarding the POM slopes (*M*_*PE*_ = -0.07, *SD*_*PE*_ = 0.17; *M*_*TE*_ = -0.08, *SD*_*TE*_ = 0.30; *t*(60) = 0.21, *p* = .83). Two participants showed extreme values (in excess of 3 SDs) in POM slopes and were thus excluded from correlation analyses involving POM slopes (i.e., POM slopes × trait empathy; POM slopes × activation slopes) to avoid a bias in the correlations due to the leverage of these data points. No correlations were observed between POM slopes and trait empathy ratings.

### Functional Data

#### Whole group analysis

**Fig 3:**
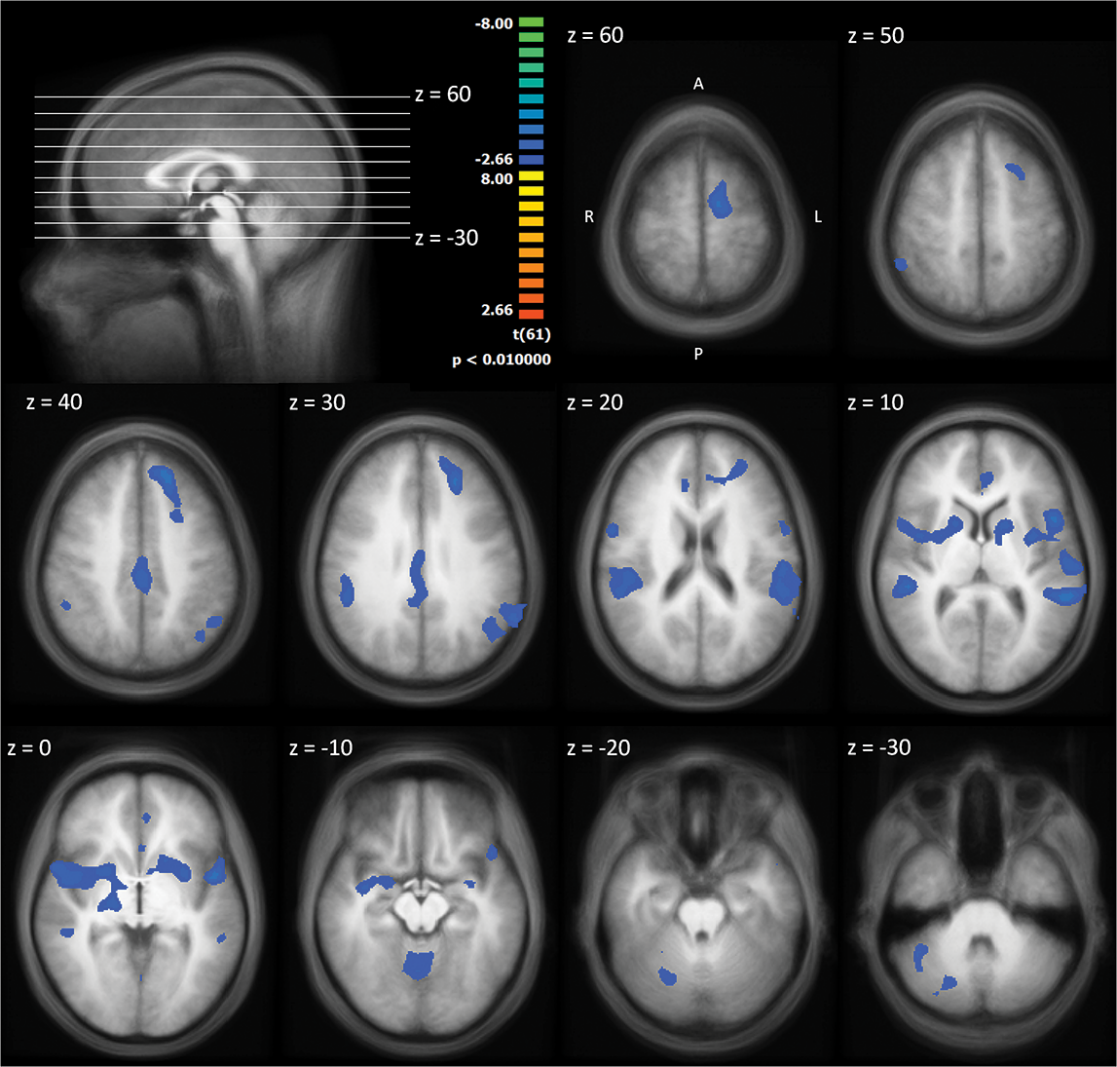
One sample t-test against zero for the slopes of the BOLD response (pain pictures > neutral pictures) across the whole sample. The statistical maps are shown superimposed on the averaged T1-weighted dataset of all subjects. Blue/green colours signify negative slopes of the BOLD response significantly different from zero, i.e. neural habituation.

We calculated a one sample *t* test against zero for the slopes of the BOLD response for the contrast pain pictures > neutral pictures across the whole sample (Fig 3, Table 2). Only clusters with negative slopes (represented by blue colour in the Figure) were found. They indicate neural habituation, The clusters’maxima were localised bilateral in the anterior/midinsular cortex (AI/MIC, Brodmann Area (BA) 13), in the cingulate gyrus (BA 31), and in the cerebellum as well as the right inferior parietal lobule (BA 40), the supplemental motor area (SMA, pre-SMA and SMA proper based on Mayka et al. [32]; BA 6), and the left superior frontal gyrus (BA 9).

**Table 2:** Cluster maxima of clusters for which the slopes of the BOLD response for the contrast pain > no pain were ≠ 0 (one sample *t*-test) across the whole sample (n = 62). All t values were negative, indicating negative slopes and thus habituation. Thresholds are based on cluster-level thresholding with an initial threshold of *p* < .01 and a cluster threshold of *p* < .05 (minimum cluster size: 1944 mm^3^).

Anatomical Description	x	y	z	*t*	Hemisphere	BA	Cluster size (mm^3^)
Inferior Parietal Lobule	53	-44	45	-4.39	R	40	8394
Insula	41	1	3	-4.34	R	13	11555
Cingulate gyrus	2	-26	36	-4.11	R	31	4967
Cerebellum, Culmen	-1	-62	-6	-3.77	L	-	4339
Medial frontal gyrus	-13	1	57	-4.52	L	6	2130
Superior frontal gyrus	-22	37	30	-4.09	L	9	10371
Insula	-46	1	3	-4.37	L	13	22781

Both right and left AI/MIC clusters extended to the inferior frontal gyrus (bilateral BA 44, left BA 47), the superior temporal gyrus (right BA 22, left BA 42), the claustrum and the putamen. On the left side, the AI/MIC cluster reached into the inferior parietal lobule (BA 39/40), the thalamus (ventral anterior nucleus, BA 8), the precentral gyrus (BA 44), the postcentral gyrus (BA 40), and the supramarginal gyrus (BA 40).

The left superior frontal gyrus cluster stretched across the pregenual anterior cingulate cortex (pACC, classified and labelled, according to Vogt [33]; BA 24) and the middle frontal gyrus (BA 8/10). The cingulate cluster (BA 31/24) included the posterior midcingulate cortex (pMCC) and the dorsal posterior cingulate cortex (dPCC) (both classified and labelled based on Vogt [33]). The cluster of the right inferior parietal lobule extended to the right superior temporal gyrus (BA 41) and the right insula (BA 13).

### Group Differences

**Fig 4:**
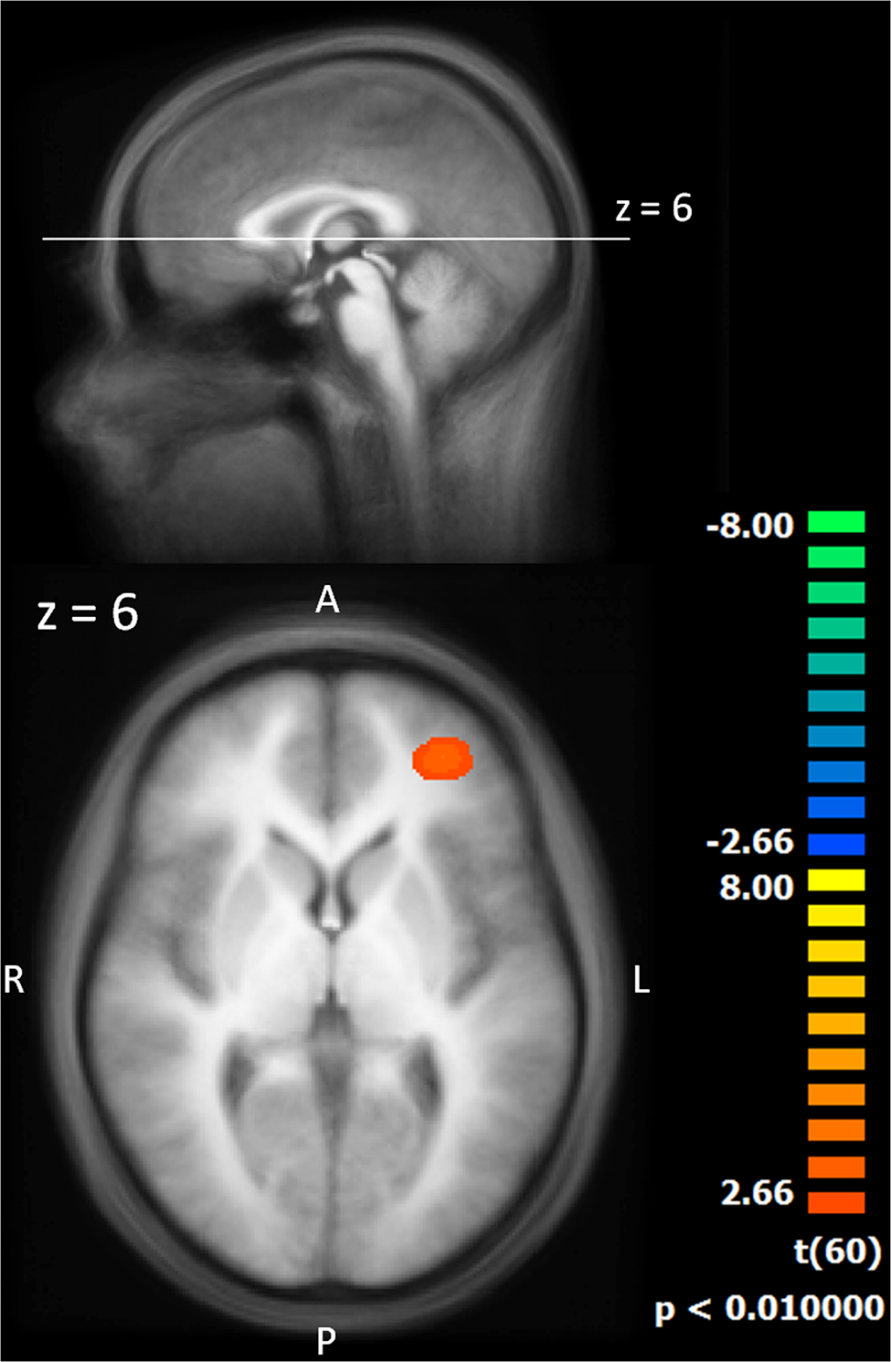
Two sample t-test between the groups’ slopes of the BOLD response (pain exposure versus touch exposure). The statistical maps are shown superimposed on the averaged T1-weighted dataset of all subjects. Yellow/orange colours signify larger values of the slopes of the BOLD response, i.e. less neural habituation.

Regarding group differences, we calculated a *t* test between the groups’ slopes of the BOLD response (pain exposure versus touch exposure) to investigate the influence of prior pain experience on habituation. The pain exposure group showed higher slope values of the BOLD response, which corresponds to less habituation in the left middle frontal gyrus (*t*(60) = 3.86; *x* = -31, *y* = 46, *z* = 6; BA 10; Cluster size = 1673 mm^3^) (Fig 4).

### Correlations

**Fig 5:**
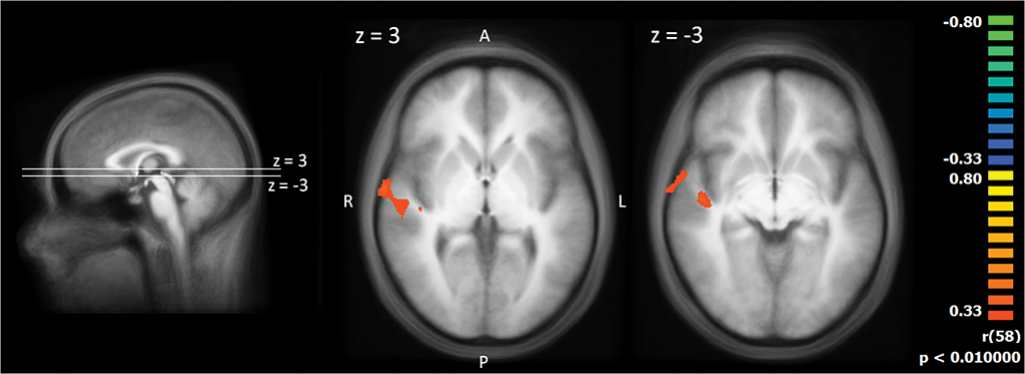
Correlation of the POM slopes with the slopes of the BOLD response. The statistical maps are shown superimposed on the averaged T1-weighted dataset of all subjects. Yellow/orange colours signify positive correlations.

**Fig 6:**
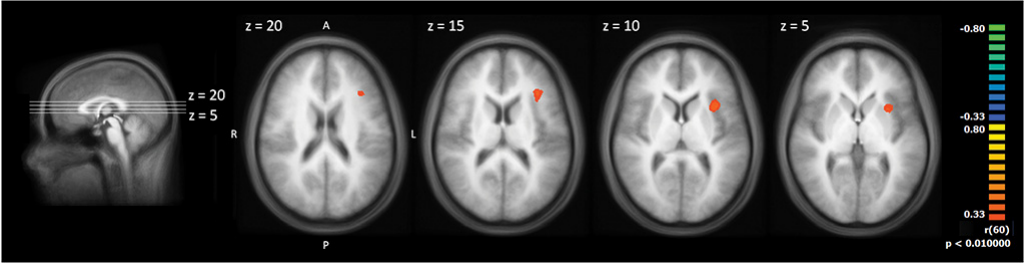
Correlation of personal distress with the slopes of the BOLD response. The statistical maps are shown superimposed on the averaged T1-weighted dataset of all subjects. Yellow/orange colours signify positive correlations.

**Fig 7:**
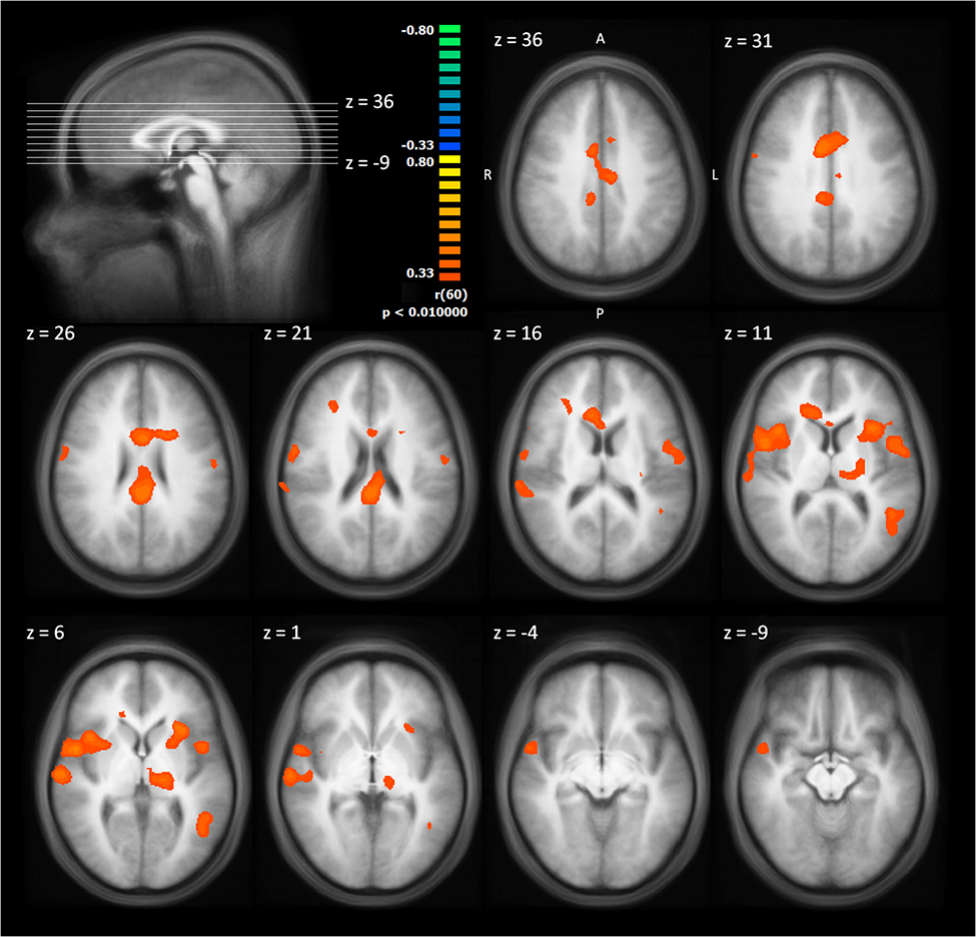
Correlation of trait fantasy with the slopes of the BOLD response. The statistical maps are shown superimposed on the averaged T1-weighted dataset of all subjects. Yellow/orange colours signify positive correlations.

**Fig 8:**
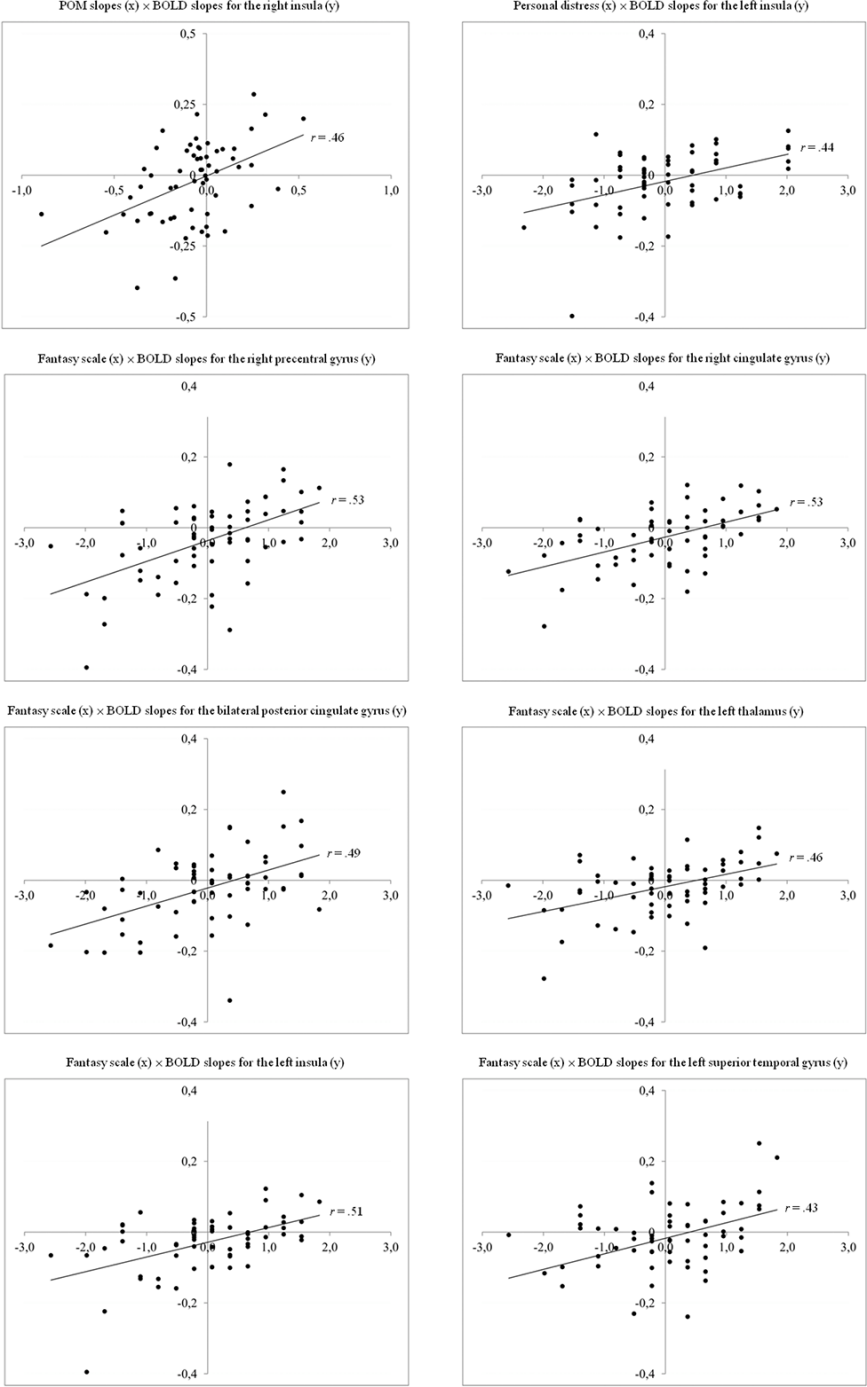
Scatterplots of correlations. Behavioural data were z-transformed. As smaller slope values of the BOLD response express stronger habituation, positive correlations between personal distress/fantasy scale and the slopes of the BOLD response indicate that a high occurrence of this trait corresponds to less neural habituation. As smaller slope values for both the BOLD response and POM ratings express stronger habituation, a positive correlation of these variables indicate that a stronger reduction in POM ratings corresponds to stronger neural habituation.

Because the groups did not differ with regard to trait empathy or POM ratings (see Behavioural Data), the data were pooled for the following correlational analyses. The POM slopes correlated positively with the slopes of the BOLD response in a cluster stretched across the right insula and superior temporal gyrus (*r* = .45; Cluster maximum *x* = 65, *y* = -8, *z* = 3; BA 22; Cluster size = 2258 mm^3^) (Fig 5). A higher reduction in pain ascribed to the model corresponds to a bigger decrement in this area. We found positive correlations between the personal distress subscale and the slopes of the BOLD response in the left insula (*r* = .44; *x* = -34, *y* = 13, *z* = 9; BA 13; Cluster size = 1326 mm^3^) (Fig 6). The higher the participants’ trait of personal distress, the less was their habituation in the left insula. There was a positive correlation of the fantasy subscale ratings and the slopes of the BOLD response in the right precentral gyrus extending to the superior temporal gyrus (BA 44) and the insula (BA 13), in the anterior cingulate cortex (BA 24/32), the posterior cingulate cortex (BA 23), the thalamus, the left insula (BA 13), and the left superior temporal gyrus (BA 39) (Fig 7, Table 3). This corresponds to less habituation in these areas in participants who tend to identify themselves with other persons. After conducting the cluster level correction, there were no significant correlations between the slopes of the BOLD response and the empathic concern and the perspective-taking subscales. The scatterplots of all correlations are illustrated in Fig 8.

**Table 3:** Cluster maxima of clusters for which the ratings of trait fantasy correlated with the slopes of the BOLD response across the whole sample (n = 62). Thresholds are based on cluster-level thresholding with an initial threshold of *p* < .01 and a cluster threshold of *p* < .05 (minimum cluster size: 1944 mm^3^).

Anatomical Description	x	y	z	*r*	Hemisphere	BA	Cluster size (mm^3^)
Precentral gyrus	50	4	9	0.53	R	44	10389
Cingulate gyrus	8	7	33	0.49	R	24	8302
Posterior cingulate	-1	-35	24	0.50	L	23	4305
Thalamus	-16	20	6	0.44	L	-	2017
Insula	-31	16	9	0.49	L	13	5392
Superior temporal gyrus	-46	-50	9	0.43	L	39	2033

## Discussion and Conclusions

The main objective of this study was to examine possible habituation effects of empathy for pain. In order to test for decrements of the neural activation over time, we reanalysed the data from our previous study [21] and found negative slopes of the BOLD response bilaterally in the insulae (AI/MIC), the cingulate gyrus (pMCC/PCC, and pACC) and SMA that partly overlap with areas typically associated with empathy for pain.

### Habituation Effects

In line with Lamm et al. [12], we did not find significant habituation effects on the rating of perceived pain (POM slopes) in response to repeated exposure to similar visual stimuli. We found negative slopes of the BOLD response in the bilateral AI/MIC, the pMCC/dPCC, the cerebellum, SMA, and the right inferior parietal lobule as well as in the left superior frontal gyrus stretching to the pACC. Our results suggest that, even though ratings of perceived pain remained at a constant level, the neural structures do not react to this pain uniformly over time, but, under certain circumstances, the initial activation decreases quickly (in our study over 40 min). A possible explanation for the discrepancy in habituation rates between the POM ratings and the neural activation might be that rating the perceived pain was regarded as a cognitive, rather than an emotional, task. It is possible that participants formed an opinion about how intense the induced pain was to the models. Once such an opinion was generated, it may be relatively stable over time and not subject to habituation. Besides, pain can be described along a sensory-discriminative dimension (pain intensity), as assessed in the present study, and an affective-motivational one (pain unpleasantness) (e.g., [34,35]). Pain unpleasantness ratings but not pain intensity ratings were influenced by dispositional empathy [36]; it would be an intriguing hypothesis for future research whether ratings of how unpleasant the pain is for the model rather than ratings of how intense the model’s pain is may be more susceptible to habituation effects.

Two recent meta-analyses [3,37] summarised the findings concerning neural correlates of empathy. Lamm and colleagues’ meta-analysis of 32 pain empathy studies indicated that a core network consisting of the bilateral anterior insular cortex and the medial-anterior cingulate cortex is associated with empathy for pain [3]. Likewise, according to Fan and colleagues’ meta-analysis of 40 empathy studies, including different emotions (e.g., pain, anxiety, and fear) and multiple tasks, the bilateral AI and a cluster centred at the aMCC extending to the dorsal ACC (dACC) and the SMA, are strongly associated with empathy [37]. We found significant habituation in the left AI/MIC, an area that corresponds well with the left insula/IFG cluster reported by Fan and colleagues [37]. The habituation cluster in the SMA approximately matched the caudal-dorsal edge of Fan and colleagues’ aMCC/dACC/SMA cluster. However, there was no spatial overlap between the habituating areas in the present study and the core network of empathy for pain proposed by Lamm and colleagues [3] as the bilateral AI/MIC and pMCC/dPCC clusters were more posterior than the areas typically linked to empathy for pain. These differences are not surprising, given the fact that fMRI studies investigating empathy for pain use repeated exposure to similar stimuli as this is a necessary precondition for brain imaging techniques in general. On the one hand, that way it is possible to investigate whether a given task reliably activates certain brain areas. On the other hand, structures that habituate over the time of the investigation are less likely to be identified using this common fMRI approach because due to the habituation the activity in the habituation areas may not show up as supra-threshold when contrasts are calculated across the full experimental time-span. The fact that the activation in these areas habituated quickly, does explain why they are less likely to be identified in studies using the summary approach. Therefore the absence of these structures in the meta-analyses does not entitle the conclusion that they do not play a role in empathy for pain.

The present data set affords the opportunity of comparing the results of the summary approach and the habituation approach. With regard to the summary approach (see [21]), the coordinates of the clusters in the aMCC and the bilateral AI are concordant with the meta-analyses [3,37]. Analysing the habituation slopes resulted in slightly posterior locations of the cluster maxima for the bilateral AI and the cingulate cortex. The question of whether any of the structures identified in our study were also initially active in some of the other studies but habituated too fast to be identified with the summary approach employed is an open one. It would be an interesting question to address in future research.

The areas that habituated over time are associated with a range of different functions. According to Vogt, the pACC is associated with coding pain unpleasantness [33] and personal relevance [38]. Enzi and colleagues [39] proposed that the pACC plays a key role in assigning affect to various types of tasks, whereas Grabenhorst and colleagues [40] concluded that the pACC is involved in cognitive modulation of affective representations. The pMCC and dPCC are involved in orienting the body in response to sensory stimuli, including nociceptive stimuli (skeletomotor orientation of the body in response to noxious stimuli) [33]. However, in all these studies, the summary approach was used. In line with the argument concerning the differences between the summary approach and the habituation approach, some caution should be exercised when the results of studies employing the summary approach are used in the interpretation of the habituation in these areas. For example, it is plausible that the pMCC/dPCC activity decreases once the initial orienting is complete and the orientation only needs to be maintained. A similar argument could be made with regard to the assigning of affect. Initially this may lead to activity in the pACC, which decreases over time as the assignment only has to be sustained.

#### Correlation of brain activity with behavioural measures

POM slopes correlated positively with activation slopes in the right superior temporal gyrus and the right insula. A higher reduction in pain ascribed to the model corresponds to a bigger decrement in these two areas, thus linking the decrease in the insula and the superior temporal gyrus directly with the pain ascribed to the model. So far, research has pointed to a connection between empathy, measured via neural correlates of empathy (e.g., [21,41,42,43]) or subjective state empathy (e.g., [19]) and the pain ascribed to the object of empathy. By correlating their habituation, we are the first to show that the neural correlates of empathy and the pain ascriptions shift in a synchronous manner.

We observed positive correlations between two subscales of trait empathy and the slopes of the BOLD response. In particular, the self-reported tendency to experience personal distress when seeing someone else in a painful or unpleasant situation was related to the slopes of the BOLD response in the left AI. The fantasy scale, i.e. the tendency to put oneself into the position of a fictional character when reading books or watching movies was associated with the slopes of the BOLD response in the bilateral AI (amongst others). As smaller slope values express stronger habituation, the higher the participants’ trait personal distress and fantasy, the weaker was their habituation in the AI. Personal distress is a self-focused, aversive affective reaction, comprising feelings of anxiety and discomfort, which results from observing another person's negative experience [26,44,45]. Tendencies towards personal distress reactions are related to a variety of psychological problems, including chronic fearfulness [46], depression [47,48,49], neuroticism [50,51], burnout [52] as well as low regulation and coping skills [53,54]. According to Mooradian and colleagues, personal distress is closely related to neuroticism, the tendency to experience negative emotions. At the heart of both lies the inability to successfully regulate one’s emotional reactions [51]. Sustained emotional reactions to another person’s suffering as observed in the present study may be one instance of the failure to down-regulate strong emotional reactions. Habituating reactions in response to repeated exposure of another person’s pain can be regarded as functional in a wide range of situations, for instance, in the case of professional health care providers who are exposed to others’ suffering on a daily basis.

Experimenters correlating neural activity with behavioural measures of trait empathy have found inconsistent results to date [55]. Whereas some studies found correlations between trait empathy scales and neural activity in the pain matrix (e.g., [36,43,56,57,58]), others have not detected any (e.g., [1,21,42,59,60]). With regard to these inconsistencies, Lamm and colleagues [3] proposed that situational rather than dispositional measures of empathy might be more sensitive and thus more likely to predict empathic neural responses. According to Bufalari and Ionta’s review [13], activity in areas coding affective qualities of observed sensations is more closely related to emotional empathy scales (i.e., empathic concern and personal distress) than activity in structures coding sensory qualities of observed sensations. The latter is differentially modulated by cognitive perspective taking and self-oriented empathic responses (i.e., personal distress). Here, we found a positive correlation of self-oriented personal distress and habituation in the AI, which extends Bufalari and Ionta’s first proposal on the context of habituation.

The correlation of trait fantasy with habituation in bilateral AI that was observed in the present data implies that this characteristic may be particularly important for maintaining neural empathic responses.

#### The influence of prior pain exposure on habituation

The POM slopes did not differ between participants with and without prior pain experience. Hence, the pain experience did not affect their course of estimating the model’s pain.

Participants with prior pain exposure showed less habituation in the left middle frontal gyrus (BA 10) than participants in the touch exposure group. According to Gilbert and colleagues [61], activation in lateral BA 10 is associated with working memory and episodic memory retrieval. Possibly participants with prior pain exposure continued to retrieve their own pain experience when viewing the pain pictures.

There were no group differences regarding the slopes of the BOLD response in regions typically associated with empathy for pain, such as AI or aMCC. Hence, our results suggest that the person’s specific prior pain experience does not influence the neural correlates for their maintenance of empathy. Contrary to our hypothesis, this finding implies that the attenuated activations in the AI and aMCC in participants with prior pain exposure in response to the pain pictures [21] was not caused by a more rapid habituation due to the exposure, but by a time invariant difference in BOLD response to pain pictures by the two groups.

### Limitations

The data were reanalysed from our previous study [21] and share some of its limitations. The participants could only see the hands of another person, whereas in real life people can use contextual cues and sensory and emotional-communicative information to infer pain in others. The visual material (showing hands instead of faces or both) limits the generalization of the results. In addition, the pictures resembled each other strongly (exclusively left hands). Possibly, diversified visual stimuli (e.g., showing various body parts) could result in a different habituation course. Another limitation is that the habituation was represented by linear gradients. In the absence of hypotheses of the course of the habituation, it seemed the most parsimonious choice, although, almost certainly, it is not the best approximation and does not take into account possible higher order polynomial courses.

The large sample with an equal distribution of men and women with and without prior pain exposure as well as including behavioural data and relating them to the functional data are clear strengths of the study.

## Conclusions

Previous research has consistently found activity in the aMCC and bilateral AI in response to the perception of another person in a painful situation. Our results complement the research on empathy by showing that repeated exposure to another person’s pain results in decreased neural activity in AI/MIC, pMCC/dPCC and SMA, even though behavioural ratings of perceived pain of the model remained at a constant level. However, although habituating areas were localized in the insula and midcingulate cortex, there is only a partial overlap with the exact areas typically reported in studies of empathy. The participant’s prior pain experience did not affect the neural maintenance of empathy. Trait measures like personal distress and fantasy modulated the neural correlates of empathy in a way that participants with high scores on these measures tended to show less habituation in the AI.

In a wide range of situations, the initial strong reaction to another person’s pain followed by habituation could be regarded as functional: a quick motivation to render assistance would be followed by attenuated responses suitable to preserving the observer’s resources, if the situation cannot be addressed within a short time frame. This mechanism may be relevant in a large variety of contexts from working in medical professions to being the partner of someone with chronic pain to depictions of pain in the media.

Our findings point to the desirability of taking into account neural habituation processes when planning studies of empathy that require a large number of repetitions.
